# Impact of moderate treadmill training on cytokine levels in rats: influence of age and sex

**DOI:** 10.3389/fragi.2026.1794896

**Published:** 2026-04-09

**Authors:** Renan F. do Espírito-Santo, Youping Zhang, Jin Y. Ro, Joyce T. Da Silva

**Affiliations:** Center to Advance Chronic Pain Research, Department of Neural and Pain Sciences, University of Maryland Baltimore, School of Dentistry, Baltimore, MD, United States

**Keywords:** aging, cytokines, exercise, inflammation, sex

## Abstract

Inflammation repairs tissues in response to stressors. Aging alters circulating pro- and anti-inflammatory markers through immunosenescence, the gradual decline of immune function, and increased cytokine secretion, contributing to chronic inflammation. Exercise can modulate cytokine levels, producing anti-inflammatory effects. However, how age and sex influence these responses remains unclear. Methods: We evaluated blood serum levels of proinflammatory (TNF-α, IL-1β, IL-6) and anti-inflammatory (IL-10) cytokines in young and old male and female Fischer 344 rats before and after a moderate treadmill exercise protocol. Results: TNF-α increased in young rats following both sedentary and exercise treatments, but not in old rats, with no sex differences. Exercise increased IL-1β and IL-6 only in young males, while old males showed elevated levels under sedentary condition that were suppressed by exercise. IL-10 increased exclusively in old females after exercise. Conclusion: These results indicate that exercise does not uniformly suppress inflammation but modulates cytokine activity in an age- and sex-dependent manner. In young rats, proinflammatory responses may support tissue repair and muscle remodeling, whereas in old rats, the attenuation of these markers, along with IL-10 elevation in females, suggests a shift toward anti-inflammatory adaptations that may counteract chronic inflammation.

## 1 Introduction

Cytokines play a vital role in regulating inflammation and maintaining immune homeostasis. As sensitive indicators of immune function, circulating cytokine levels serve as valuable biomarkers for assessing overall health. Elevated levels of pro-inflammatory cytokines, such as interleukin-6 (IL-6), tumor necrosis factor-alpha (TNF-α), and interleukin-1 beta (IL-1β), have been consistently linked to an increased risk of chronic diseases, including cardiovascular disorders, metabolic syndrome, and neurodegenerative conditions (Haybar et al., 2023; Na et al., 2014; Popa et al., 2007). In contrast, anti-inflammatory cytokines like interleukin-10 (IL-10) exert immunoregulatory effects, promoting tissue repair and maintaining immune balance (Ouyang and O'Garra, 2019; Saraiva and O'Garra, 2020). The intricate balance of cytokine production is crucial for maintaining health, and disruptions in this balance have been implicated in various disease states.

Aging is characterized by a decline in physical function, increased risk of chronic diseases, and altered physiological processes, including immune response and inflammatory regulation (Cisneros et al., 2022). A key contributor to this decline is “inflammaging,” a chronic, low-grade increase in pro-inflammatory cytokines, such as IL-6 and TNF-α, that accumulates over time (Ajoolabady et al., 2023). This age-related shift in cytokine balance is thought to result from a combination of factors, including cellular senescence, mitochondrial dysfunction, and impaired immune regulation (Coperchini et al., 2025). However, the relationship between aging and cytokine levels is complex, and some studies have found no significant associations (Ahluwalia et al., 2001; Beharka et al., 2001; Kim et al., 2011). This discrepancy may be due to the fact that aging is often accompanied by various disease conditions and treatments that can influence cytokine profiles. As a result, it can be challenging to distinguish between changes in cytokine levels caused by aging itself versus those caused by age-related health conditions in elderly populations.

Exercise is a critical component of healthy aging, mitigating the risk of chronic diseases and exerting anti-inflammatory effects through modulation of immune function (Huntula et al., 2022). A substantial body of evidence demonstrates that moderate to strenuous exercise and regular physical activity significantly reduce inflammatory markers in older adults (Beavers et al., 2010a; Colbert et al., 2004; Kohut et al., 2006; Reuben et al., 2003; Taaffe et al., 2000). Research using animal models, such as laboratory rats, has shown that exercise can modulate the immune response, decrease systemic inflammation, and promote a more favorable cytokine profile (Walsh et al., 2011a; Walsh et al., 2011b). Most preclinical research investigating the effects of exercise on cytokines has used male rodents and the impact of moderate exercise on circulating cytokines in aging rats, particularly regarding potential sex differences, remains unclear. Further research is needed to elucidate the specific types of exercise that optimize aging-related cytokine responses, particularly in homogeneous populations controlled for age, sex, and disease status.

This study examines the effects of moderate treadmill exercise on blood cytokine levels in young and old, male and female rats. We aim to elucidate how age and sex influence the inflammatory response to physical activity and explore the potential benefits of exercise in mitigating age-related inflammation. By comparing these groups, we seek to provide valuable insights into the immune response to exercise and identify potential sex-specific differences.

## 2 Results

### 2.1 Impact of age and sex on exercise-induced changes in cytokines

#### 2.1.1 TNF-α

**FIGURE 1:**
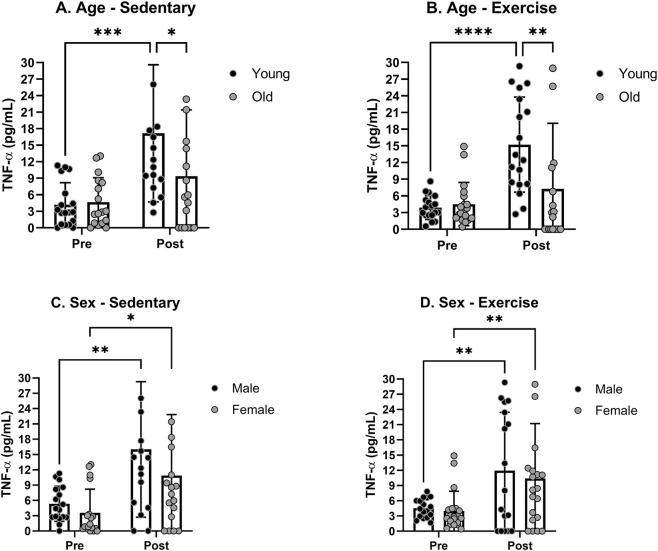
Blood serum TNF-α levels pre- and post-moderate exercise regime. Graphs show **(A)** sedentary aged groups, **(B)** exercise aged groups, **(C)** sedentary groups stratified by sex, and **(D)** exercise groups stratified by sex. All the results represent S.E.M. and n = 8–10 rats per group, with the p values obtained from statistical analyses (*p < 0.05, **p < 0.005, ***p < 0.0005, ****p < 0.0001).

We initially assessed the impact of age on exercise-induced changes in blood cytokine levels by comparing young versus old rats, regardless of their sex. The mean levels of TNF-α prior to any treatments were not significantly different between young and old rats in either the sedentary or exercise groups. There was a significant increase in the mean TNF-α levels in young rats following the treatment in both sedentary and exercise groups (Figures 1A,B). Although there was a slight increase in the TNF-α level in old rats, the increase did not reach statistical significance. A *post hoc* analysis revealed a significant age effect after the treatment in both sedentary and exercise groups. The impact of sex on exercise-induced changes in blood TNF-α levels was assessed by comparing male versus female rats, regardless of their age (Figures 1C,D). The mean levels of TNF-α prior to any treatments were not significantly different between male and female rats in either the sedentary or exercise groups. The TNF-α level increased significantly in both male and female rats under both sedentary and exercise conditions. A *post hoc* analysis revealed no significant sex effect after the treatment in both sedentary and exercise groups.

**FIGURE 2:**
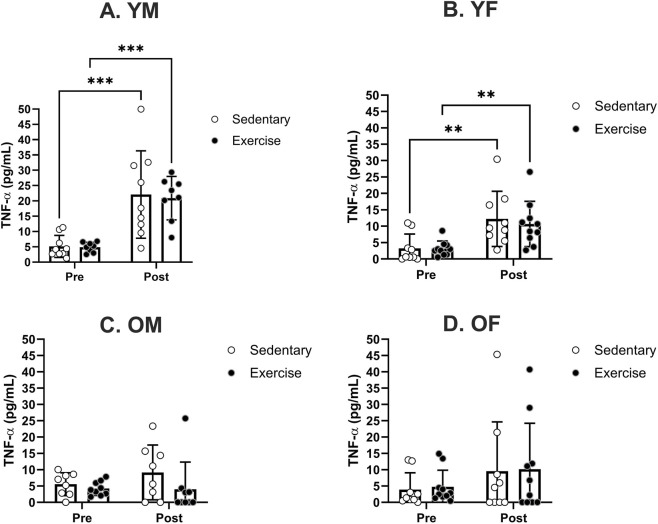
Specific group analyses showing blood serum TNF-α levels pre- and post-moderate exercise regime. Graphs show the effects of sedentary and exercise regimes for each group: **(A)** young males, **(B)** young females, **(C)** old males, and **(D)** old females. All the results represent S.E.M. and n = 8–10 rats per group, with the p values obtained from statistical analyses (**p < 0.005, ***p < 0.0005).

To enhance clarity regarding the treatment’s impact on blood TNF-α levels, we conducted additional analysis by categorizing the samples based on each sex and age group (Figure 2). There was no significant difference in pre-treatment TNF-α levels in any of the sex and age groups. TNF-α levels were significantly increased following the treatment in YM and YF groups (Figures 2A,B), but not in OM and OF groups (Figures 2C,D). Post-hoc analyses revealed no significant treatment effect in any of the sex and age groups.

#### 2.1.2 IL-1β

**FIGURE 3:**
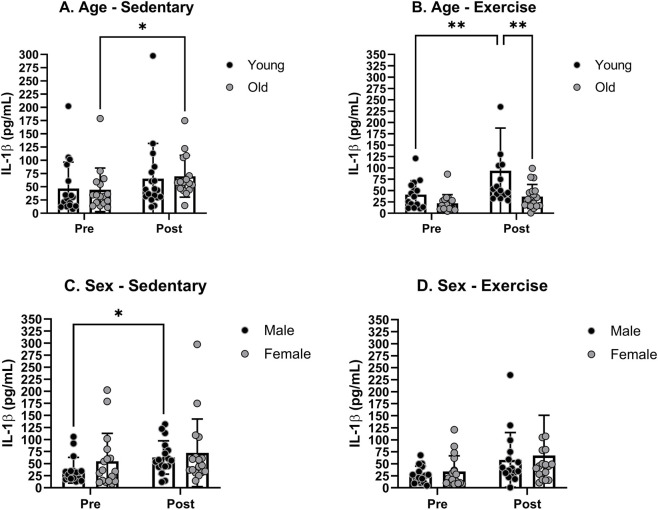
Blood serum IL-1β levels pre- and post-moderate exercise regime. Graphs show **(A)** sedentary aged groups, **(B)** exercise aged groups, **(C)** sedentary groups stratified by sex, and **(D)** exercise groups stratified by sex. All the results represent S.E.M. and n = 8–10 rats per group, with the p values obtained from statistical analyses (*p < 0.05, **p < 0.005).

The mean levels of IL-1β prior to any treatments were not significantly different between young and old rats in either the sedentary or exercise groups. There was a slight increase in the mean IL-1β levels in sedentary groups (Figure 3A). The increase was more profound in the exercise group, with a significant increase only in young rats (Figure 3B). A *post hoc* analysis revealed a significant age effect in the exercise groups. The mean levels of IL-1β prior to any treatments were not significantly different between male and female rats in either the sedentary or exercise groups. The IL-1β level increased slightly in both male and female rats under both sedentary and exercise conditions (Figures 3C,D). A *post hoc* analysis revealed no significant sex effect after the treatment in both sedentary and exercise groups.

**FIGURE 4:**
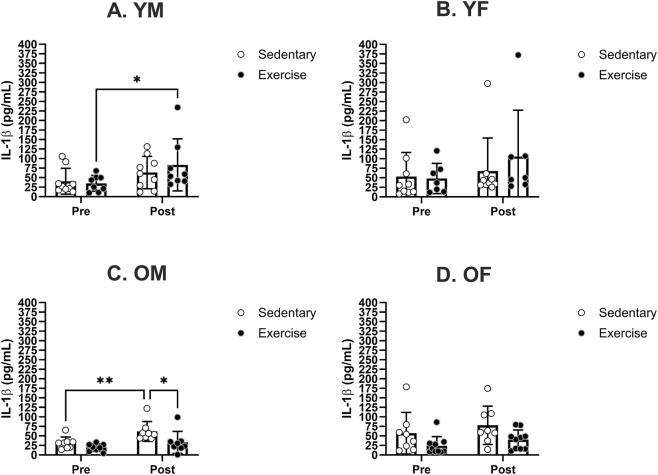
Specific group analyses showing blood serum IL-1β levels pre- and post-moderate exercise regime. Graphs show the effects of sedentary and exercise regimes for each group: **(A)** young males, **(B)** young females, **(C)** old males, and **(D)** old females. All the results represent S.E.M. and n = 8–10 rats per group, with the p values obtained from statistical analyses (*p < 0.05, **p < 0.005).

Our analysis of blood IL-1β levels in each sex and age group revealed no significant difference in pre-treatment IL-1β levels among any of the sex and age groups. The treadmill exercise significantly increased the mean IL-1β level in YM (Figure 4A). The mean IL-1β level was significantly increased in OM following sedentary treatment but not following the exercise treatment (Figure 4C). A *post hoc* analysis revealed a significant treatment effect in the OM group. There were no significant changes in the IL-1β levels in either YF or OF group (Figures 4B,D).

#### 2.1.3 IL-6

**FIGURE 5:**
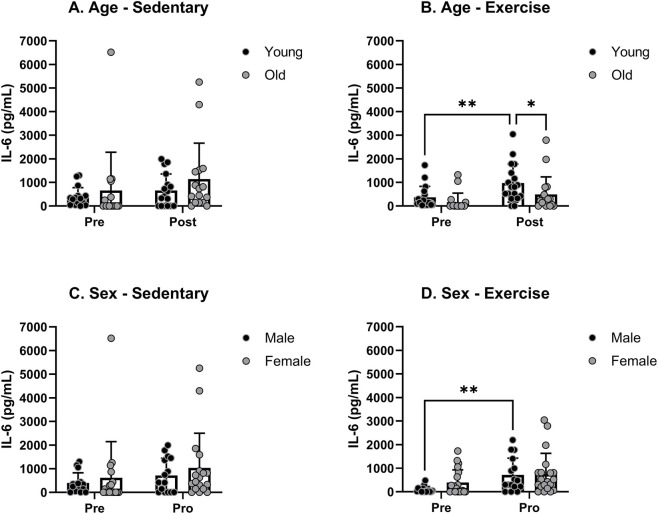
Blood serum IL-6 levels pre- and post-moderate exercise regime. Graphs show **(A)** sedentary aged groups, **(B)** exercise aged groups, **(C)** sedentary groups stratified by sex, and **(D)** exercise groups stratified by sex. All the results represent S.E.M. and n = 8–10 rats per group, with the p values obtained from statistical analyses (*p < 0.05, **p < 0.005).

The baseline IL-6 levels showed no significant difference between young and old rats in both sedentary and exercise groups before any treatments were administered. There was a significant age effect in exercise groups, but not in sedentary groups (Figures 5A,B). The increase was significant only in young rats. A *post hoc* analysis confirmed a significant age effect following the exercise treatment. The mean levels of IL-6 prior to any treatments were not significantly different between male and female rats in either the sedentary or exercise groups. There was no significant sex effect in either the exercise or sedentary groups (Figures 5C,D). Exercise treatment significantly increased the IL-6 level only in male rats, but a *post hoc* analysis revealed no significant sex effect after the treatment in both sedentary and exercise groups.

**FIGURE 6:**
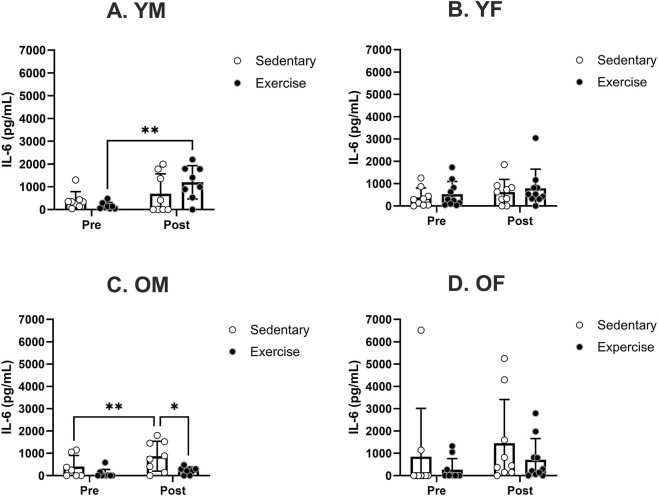
Specific group analyses showing blood serum IL-6 levels pre- and post-moderate exercise regime. Graphs show the effects of sedentary and exercise regimes for each group: **(A)** young males, **(B)** young females, **(C)** old males and **(D)** old females. All the results represent S.E.M. and n = 8–10 rats per group, with the p values obtained from statistical analyses (*p < 0.05, **p < 0.005).

The changes in IL-6 levels displayed comparable patterns to IL-1β. No notable distinctions were observed in the IL-6 levels before treatment among any of the specified sex and age groups (Figure 6). Similar to IL-1β, treadmill exercise significantly increased the mean IL-6 level in YM (Figure 6A). In OM, the mean IL-6 level significantly increased after sedentary treatment but not following the exercise treatment (Figure 6C). Post-hoc analysis revealed a significant treatment effect in the OM group. There were no significant changes in the IL-1β levels in either the YF or OF group (Figures 6B,D).

#### 2.1.4 IL-10

**FIGURE 7:**
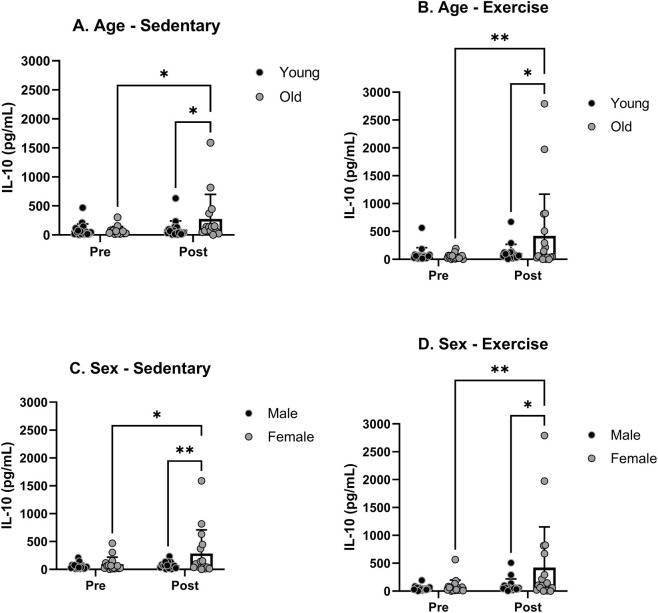
Blood serum IL-10 levels pre- and post-moderate exercise regime. Graphs show **(A)** sedentary aged groups, **(B)** exercise aged groups, **(C)** sedentary groups stratified by sex and **(D)** exercise groups stratified by sex. All the results represent S.E.M. and n = 8–10 rats per group, with the p values obtained from statistical analyses (*p < 0.05, **p < 0.005).

Finally, we examined the variations in the blood levels of IL-10, serving as a representative anti-inflammatory cytokine. Initially, there was no significant difference in baseline IL-10 levels between young and old rats in either the sedentary or exercise groups before any treatments were administered. However, a significant age effect was observed in both the sedentary and exercise groups (Figures 7A,B). In contrast to other cytokines, the increase was notable only in old rats. Post-hoc analysis confirmed a significant age effect following both sedentary and exercise treatments. The baseline levels of IL-10 were not significantly different between male and female rats in either the sedentary or exercise groups before any treatments (Figures 7C,D). However, there was a significant sex effect in both the exercise and sedentary groups. The increase in IL-10 levels was observed only in female rats for both sedentary and exercise groups. Post-hoc analysis revealed a significant sex effect after treatment in both sedentary and exercise groups.

**FIGURE 8:**
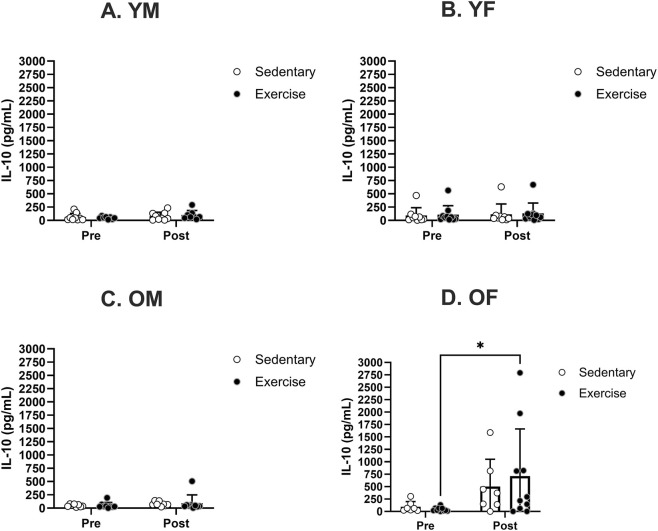
Specific group analyses showing blood serum IL-10 levels pre- and post-moderate exercise regime. Graphs show the effects of sedentary and exercise regimes for each group: **(A)** young males, **(B)** young females, **(C)** old males, and **(D)** old females. All the results represent S.E.M. and n = 8–10 rats per group, with the p values obtained from statistical analyses (*p < 0.05).

No notable differences in IL-10 levels were found before treatment among any of the specified sex and age groups (Figure 8). IL-10 levels were not significantly altered in either sedentary or exercise groups in YM, YF, OM groups (Figures 8A–C). However, the IL-10 level was significantly increased in OF following the exercise treatment (Figure 8D).

## 3 Discussion

Our study yielded several notable findings. Firstly, baseline levels of the four analyzed cytokines (TNF-α, IL-1β, IL-6, and IL-10) did not differ significantly between young and old rats or between male and female rats. Secondly, TNF-α levels increased significantly after both sedentary and exercise treatments in young rats, but not in old rats, with no sex-based differences. Thirdly, exercise induced significant elevations in IL-1β and IL-6 levels exclusively in young male rats, while the same cytokines were elevated in old male rats under sedentary conditions, which was blocked by exercise; similar age-related and sex-related change patterns observed for both cytokines. Lastly, IL-10 levels increased significantly only in old female rats following exercise. Our study compared responses at a matched absolute workload rather than at equivalent relative intensity. All rats performed the same externally imposed task (identical speed, incline, and duration), ensuring equal mechanical and metabolic demand across groups and allowing observed differences to more directly reflect intrinsic age- and sex-related physiological variation. Although this design resulted in different relative intensities across groups due to differences in aerobic capacity, this was an expected and biologically meaningful consequence; future studies using protocols standardized to relative intensity may provide complementary insight into exercise-induced cytokine responses.

Previous human studies on the impact of aging on circulating cytokine levels have yielded conflicting results. While a substantial body of evidence links inflammatory cytokine levels to increasing age, many of these studies have notable limitations, including poorly controlled health status, small sample sizes, and unaccounted environmental influences (e.g., smoking and drinking). These factors can act as confounding variables, particularly in elderly populations with multiple health issues. For instance, a study by Ferrucci et al. (Ferrucci et al., 2005) found that adjusting for cardiovascular risk factors and morbidity substantially reduced the effect of age on multiple proinflammatory cytokine levels. Other human studies that more stringently controlled for age and health status, and analyzed extensive cytokine and chemokine profiles, reported no significant association between TNF-α, IL-6, and IL-1β levels and age (Kim et al., 2011). These findings are consistent with other research reporting unchanged levels of blood cytokines with aging in healthy subjects (Ahluwalia et al., 2001; Beharka et al., 2001). The use of laboratory rats as a model system mitigate the impact of environmental factors and underlying diseases. By examining relatively homogeneous groups of rats born and raised in similar environments, we found no significant differences in basal circulating cytokine levels between young and old, or male and female, rats. Our results suggest that increases in circulating cytokine levels may be more closely associated with age-related health conditions rather than aging itself. While a systemic proinflammatory state is a significant risk factor for various aging-related conditions, (Cisneros et al., 2022), large-scale human studies with diverse populations and stringent control for environmental and lifestyle factors are needed to establish a clearer picture of blood cytokine levels as reliable biomarkers in the elderly population.

Physical exercise can affect multiple body systems. We have previously shown that moderate exercise can improve endogenous pain-inhibitory responses in young animals, even in the absence of changes in testosterone levels (do Espírito Santo et al., 2024). It is also known that regular physical activity can alter the production and circulation of various cytokines, including both pro-inflammatory and anti-inflammatory types via the nuclear factor κB (Suzuki et al., 2020) and Toll-like receptors (Gleeson et al., 2011) pathways. This exercise-induced shift in cytokine balance can have significant implications for overall health, potentially reducing chronic inflammation and improving immune function (Nishida et al., 2014; Palmefors et al., 2014). For the elderly population, all types of exercise can be beneficial when tailored to individual factors and limitations; however, a progressive low-intensity aerobic exercise program is particularly recommended (Huntula et al., 2022). Aerobic exercise modulates inflammatory cytokines and immune responses more effectively than resistance exercise training in the elderly (Abd El-Kader and Al-Shreef, 2018). Furthermore, a moderate intensity paradigm is more clinically relevant for older adults; many older adults are not able to exercise at an intense pace. An exercise paradigm of 30 min of moderate exercise 5 days a week is consistent with the activity recommendations from the American Heart Association, the United States Department of Health and Human Services, and the World Health Organization (Nelson et al., 2007; Oja and Titze, 2011). Thus, it is important to understand the effects that this level of activity may have on the circulating cytokine levels.

Based on our findings, the pronounced rise in TNF-α observed in young but not old rats following both sedentary handling and exercise may indicate that younger animals can be more susceptible to a strong, possibly maladaptive, acute inflammatory response to environmental stress. In contrast, the absence of such reactivity in aged rats could reflect immunosenescence, characterized by attenuation of dynamic immune responses despite a background of low-grade inflammation. In support of this, Bektas et al. describe how aging typically narrows the amplitude of acute inflammatory responses, even when baseline cytokine levels remain elevated (Bektas et al., 2017). Furthermore, innate immune cells such as macrophages become increasingly dysfunctional with age—displaying reduced TNF-α secretion upon stimulation—while simultaneously contributing to systemic inflammation (Ongrádi and Kövesdi, 2010). Interestingly, physical stress, such as the treadmill handling aspect, even without actual exercise, has been shown in aged rodent models to reduce basal inflammatory cytokines, including TNF-α and IL-1β, possibly by engaging counter-regulatory pathways (Wu et al., 2022).

IL-1β and IL-6 exhibited similar patterns, showing significant increases solely in young male rats following exercise. This pronounced proinflammatory response in young males may be linked to the effects of sex hormones on immune activation (Gholamnezhad et al., 2014), as well as the heightened metabolic demands associated with physical activity (Pedersen et al., 2004; Pedersen et al., 2001). Specifically, IL-6 is a myokine released by skeletal muscle during contraction. It plays a crucial role in substrate mobilization and energy metabolism, particularly during periods of high metabolic demand (Kistner, Pedersen and Lieberman, 2022; Pedersen and Febbraio, 2012). The increased IL-6 response observed in young males likely reflects its dual function as a cytokine and metabolic signal during exercise. In contrast, older males exhibited elevated cytokine levels under sedentary conditions, which were attenuated by moderate physical activity. These findings are consistent with previous reports, which have shown that long-term moderate exercise reduces basal systemic inflammation in aging subjects, as evidenced by decreased levels of IL-6 and other proinflammatory markers following structured exercise interventions (Beavers et al., 2010b; El Assar et al., 2022).

Beyond serving as circulating inflammatory markers, TNF-α, IL-1β, IL-6, and IL-10 directly regulate skeletal muscle turnover and regenerative capacity. TNF-α and IL-1β activate NF-κB signaling and ubiquitin–proteasome pathways, promoting proteolysis and impairing myogenic differentiation when chronically elevated, thereby contributing to anabolic resistance and sarcopenia (Collins and Grounds, 2001; Li, 2003). In our study, the robust increase in TNF-α, IL-1β, and IL-6 observed exclusively in young males following exercise likely reflects a transient, adaptive inflammatory response that supports muscle remodeling and metabolic signaling rather than catabolism. IL-6, in particular, acts as a contraction-induced myokine that enhances substrate mobilization and may facilitate satellite cell activation during acute exercise (Belizário et al., 2016; Forcina et al., 2022). In contrast, the absence of this response in aged males, together with elevated cytokine levels under sedentary conditions, suggests a shift toward a chronic low-grade inflammatory milieu characteristic of aging, which may blunt anabolic signaling and impair hypertrophic responsiveness.

Notably, females exhibited a distinct cytokine pattern. The lack of significant exercise-induced increases in pro-inflammatory cytokines in young females may reflect estrogen-mediated modulation of NF-κB activity and enhanced inflammatory resolution, limiting excessive proteolytic signaling (Straub, 2007). Furthermore, the selective elevation of IL-10 in old females following exercise indicates a sex-specific enhancement of anti-inflammatory pathways. IL-10 promotes macrophage polarization toward a pro-regenerative phenotype and suppresses TNF-α and IL-1β production, processes essential for effective muscle repair and preservation of muscle mass (Saraiva and O’Garra, 2010). Collectively, these findings suggest that while young males exhibit a strong acute pro-inflammatory remodeling response, aged females preferentially activate anti-inflammatory mechanisms, highlighting sex-dependent differences in how exercise modulates muscle–immune crosstalk during aging.

The absence of significant proinflammatory cytokine changes in females may reflect the modulatory effects of estrogen on immune responses. Estrogen has been shown to exert anti-inflammatory effects by regulating immune cell activity and accelerating the resolution of inflammation, which could explain the weakened cytokine response observed in females (Straub, 2007).

The selective increase in IL-10 following exercise in older females is particularly significant. IL-10 is a crucial anti-inflammatory cytokine that facilitates immune regulation and tissue repair (Iyer and Cheng, 2012). Its elevation may represent a compensatory mechanism in aging women, increasing resistance against low-grade chronic inflammation. This finding suggests that older women may derive unique immunoregulatory benefits from moderate exercise compared to men, a pattern consistent with sex-dependent differences reported in human studies (Islam et al., 2022). Exercise-induced IL-10 elevation helps moderate inflammatory responses and promotes the resolution of inflammation, thereby contributing to immune homeostasis (Ostrowski et al., 1999). IL-10 also inhibits the production of pro-inflammatory cytokines such as TNF-α and IL-1β, highlighting its central role in also modulating acute exercise-induced inflammation (Małkowska and Sawczuk, 2023).

Taken together, these data highlight that exercise does not uniformly suppress inflammation, but rather dynamically modulates cytokine activity, depending on age and sex. In young rats, the transient increase in proinflammatory cytokines may be adaptive, facilitating tissue repair, muscle remodeling, and metabolic regulation (Tu and Li, 2023). In older rats, the absence or attenuation of these increases, together with an elevation of IL-10 in females, points to a shift toward anti-inflammatory adaptations that may counteract inflammation (Tayebi et al., 2025; Woods et al., 2012).

This study had some limitations which reduce the weight of the inferences drawn from these findings. Exercise protocols in rodents usually rely on an imperative stimulus, typically a noxious electric shock, to facilitate adherence. Although this allows precise control of activity, it may increase stress and potentially confound pain outcomes and cytokine levels (Himmerich et al., 2013). To address this, we have previously quantified the number of shocks received on the first training day of each week (do Espírito Santo et al., 2024). Although old male and female rats received more shocks than young females during the first week, no group differences were observed in subsequent weeks, suggesting minimal confounding from noxious stimulation. Consistently, serum corticosterone levels measured at baseline and after the exercise program showed no time-related changes in any group (do Espírito Santo et al., 2024). It is worth mentioning that although our previous study demonstrated that moderate exercise does not alter testosterone levels (do Espírito Santo et al., 2024), endogenous testosterone may still play an important role in modulating cytokine and myokine release and inflammation. A previous study found that increased levels of IL-12– and IL-1β were observed in males compared with females both *in vivo* and *in vitro*, and that this effect was dependent on testosterone, suggesting that androgen exposure can upregulate specific pro-inflammatory cytokines (Posma et al., 2004). In contrast, other studies suggested that testosterone may have anti-inflammatory effects; however, the magnitude and conditions required for this action remain debated (Mohamad et al., 2019). Specifically, regarding cytokines released by skeletal muscle (i.e., myokines), testosterone has been shown to activate distinct *in vitro* intracellular pathways in male and female muscle cells (Sgrò et al., 2024; 2025). For example, female muscle cells convert testosterone to estradiol, resulting in reduced release of IL-6 and IL-8. In contrast, male muscle cells exhibited higher levels of these myokines (Sgrò et al., 2024).

Thus, future research should aim to explore the longitudinal dynamics of cytokines and myokines, evaluate additional immune markers, and directly investigate the mechanisms underlying the observed sex- and age-dependent effects. Studies that incorporate various exercise methods and intensities could provide a more comprehensive understanding of how different training routines influence inflammation regulation throughout life. Age-related alterations in cytokine kinetics may contribute to the observed patterns. Future studies incorporating higher temporal resolution sampling would help definitively disentangle these mechanisms. In summary, our research reveals that moderate treadmill exercise has distinct effects on circulating cytokine levels in rats, which vary by age and sex. Whereas young males exhibit robust increases in proinflammatory markers, old males benefit from exercise-mediated attenuation of IL-1β and IL-6, and old females uniquely show an increase in the anti-inflammatory cytokine IL-10. These findings provide new insights into the complex interaction between physical exercise, age, and sex in the shaping of immune function, highlighting the importance of personalized approaches when considering physical exercise as a strategy to promote healthy aging.

## 4 Materials and methods

### 4.1 Animals

We obtained both young (3–6 months old) and old (20–24 months old) male and female Fischer 344 rats from the National Institute of Aging. These rats were kept in a room with controlled temperature and a 12-h light-dark cycle, and they had unrestricted access to food and water. Throughout the research, continuous monitoring was implemented to minimize any unnecessary stress or discomfort for the rats. All procedures were carried out in accordance with the guidelines specified in the National Institutes of Health Guide for the Care and Use of Laboratory Rats, as well as the standards set by the International Association for the Study of Pain. Furthermore, these procedures adhered to the approved protocols of the Institutional Animal Care and Use Committee at the University of Maryland. The rats were given a minimum of 7 days to acclimate in the housing facility before the initiation of the study.

### 4.2 Treadmill exercise protocol

Age-matched male and female rats of were randomly assigned to either the exercise or sedentary group. The groups included young males engaged in exercise (YME), sedentary young males (YMS), young females in the exercise group (YFE), sedentary young females (YFS), old males engaged in exercise (OME), sedentary old males (OMS), old females in the exercise group (OFE), and sedentary old females (OFS). Rats in the exercise group were provided access to a motorized treadmill (Columbus Instruments, Columbus, OH, USA). The treadmill exercise routine involved running at a speed of 6 m/min for 30 min daily for the first week, followed by running at a speed of 12 m/min for 30 min daily for the subsequent 5 weeks. The exercise sessions occurred from Monday to Friday each week, excluding weekends. Each running lane featured an electrical stimulus system with either three or six shock grids. If a rat failed to maintain the designated speed, it would fall onto the shock grids, receiving a 200-millisecond pulse of a single electric shock with a current of 0.5 mA. The treadmill was set at a 0° inclination. In the sedentary group, rats were placed in the treadmill lanes for an equivalent duration (30 min daily) and the same total period (6 weeks), but the treadmill machine remained inactive during this time.

### 4.3 Blood cytokine analysis

Blood samples were collected from the artery on the ventral side of the rat’s tail before initiating the exercise routine and after its completion. The rats were anesthetized with isoflurane (1.5%–2%) during all blood collection procedures. These samples were obtained between 12 p.m. and 3 p.m., then centrifuged to isolate the serum, and subsequently stored at −20 °C until the day of the assay. The Luminex multi-analyte profiling (xMAP) technology from Luminex Corp. was employed to assess cytokine levels. Bead sets were coated with capture antibodies specific to various cytokines (TNF-α, IL-1β, IL-6, and IL-10). Subsequently, fluorescence detection antibodies were applied to bind the cytokine-capture antibody complex on the bead set. The distinct bead sets with fluorogenic emission detection, as identified through flow cytometric analysis, enabled the recognition of multiple cytokines in the samples. Each sample underwent triplicate measurements.

### 4.4 Statistical analyses

Statistical analysis of cytokine levels was conducted using GraphPad Prism nine software. Two-Way Repeated Measures ANOVA was executed to identify significant treatment and time effects within each age and sex group, whether sedentary or exercised. After comparisons involving multiple groups, a *post hoc* Bonferroni test was applied. Statistical significance was established at *p* < 0.05, and the data were expressed as mean ± standard error of the mean (S.E.M.).

## Data Availability

The raw data supporting the conclusions of this article will be made available by the authors, without undue reservation.
